# A cross-sectional study of the association between mobility test performance and personality among older adults

**DOI:** 10.1186/s12877-016-0272-8

**Published:** 2016-05-18

**Authors:** Maayan Agmon, Galit Armon

**Affiliations:** Department of Nursing, Faculty of Social Welfare and Health Sciences, University of Haifa, Haifa, 31905 Israel; Department of Psychology, University of Haifa, Haifa, 31905 Israel

**Keywords:** Mobility, Timed-up and go, Personality, Dual-task, Older adults

## Abstract

**Background:**

Falls among the elderly are a major public health challenge. The Timed-Up and Go (TUG) test is commonly used to identify older adults with mobility limitations. This study explored the association between TUG test results and personality among community-dwelling older adults.

**Methods:**

This cross-sectional study included 85 older adults. Personality was evaluated with the Five Factor Model. Times to complete the TUG as a single task (TUGST) alone and also with an additional cognitive task i.e., dual-task (DT), were recorded. Ordinary least squares OLS regression models were used to examine the associations between personality factors and both single DT TUG.

**Results:**

Extraversion was found to be inversely associated with time to complete the TUGST (β = -.26, *p* < .05). Conscientiousness was inversely associated with TUGDT (β = -.24, *p* < .01).

**Conclusions:**

Findings from this study highlight the relationship between personality and the TUG test. Specifically, older adults with high Extraversion completed the TUGST test more quickly than those who had lower measures of this trait and, people with high Conscientiousness completed the TUGDT tests more quickly. These findings may contribute to early identification of older adults at higher risk from mobility limitations and falls, and to developing personality-tailored interventions for fall prevention.

## Background

Falls are a major concern among the elderly and have aroused significant public health interest in recent years: [1] Falls cause approximately 40 % of all injury deaths among older adults, [2] 81–98 % of hip fractures [3], and they may dramatically alter individual’s health status and quality of life in many ways such as increase anxiety, depression, decrease social participation and increase isolation [4]. Costs resulting from falls range between 64.4 - 85.4 billion dollars in the US per-year [4].

Early detection of mobility limitations may lead to prompt and effective interventions. In recent years, a growing body of literature has investigated the association between personality and aging [5–7]. These studies demonstrated that personality is a useful tool to predict longevity and quality of life. Overall, one of the traits of the Five Factor Model of personality, Conscientiousness [8], is considered as a protective trait against age related deterioration in many aspects of life. The findings are mixed regarding other traits. Moreover, these studies addressed the potential contribution of aspects of personality towards personalized and tailored medicine to prevent age related deterioration [5–7]. Since mobility is a significant element determining the quality of life of the elderly, understanding its association with personality factors may lead to better detection of impairments and interventions. Several studies sought to understand the relationship between personality factors and mobility among the elderly, most of which used the Five Factor Model (FFM) to measure personality [6, 9, 10]. The FFM classifies most personality traits under one of five dimensions: Extraversion (e.g., bold and extraverted), Agreeableness (e.g., cooperative and kind), Conscientiousness (e.g., organized and practical), Neuroticism (e.g., moody and touchy), and Openness to experience (e.g., creative and intellectual) [8]. Many of these studies assessed mobility based on self-reports [6, 11] or muscle strength measurements, which are only indirectly associated with mobility [10]. One study measured the association between gait speed and personality and found that higher Conscientiousness, but not Openness, was associated with faster initial gait speed and with less decline in gait speed over a three-year period [9]. It did not measure the effect of other personality traits such as Extroversion, Neuroticism and Agreeableness on gait speed.

Although studies on the association between personality and aging are increasing, research on the connection between personality and mobility is in its infancy. Since Timed-up and Go (TUG) is a frequently used measure to detect mobility limitations among the elderly [12, 13], understanding how its performance is related to personality factors might contribute to the understanding of mobility deterioration in the elderly, as well as to the development of interventions tailored to personality type for people at risk of falls. Previous studies found a relationship between personality traits and some aspects of mobility (e.g. walking speed and disability) [9, 11]. The theoretical explanation for these relationships relies mostly on health behaviours that are associated with a specific trait. For example, older adults with high conscientiousness, are more engaged in physical activities compared to their counterparts, and thus are more likely to be more resilient to walking deterioration [9–11].

The TUG test is recommended by both the American Geriatric Society and the British Geriatric Society [14] among others, as a screening tool for fall risk. The TUG test includes standing, turning and sitting movements that comprise an integral part of daily mobility activities [13, 15, 16]. This test can be conducted as a single task and with additional tasks (i.e. subtraction by three or carrying a tray) as a dual-task (TUGDT). The TUG test is highly correlated with many outcomes that are critical for daily function, such as gait speed and functional mobility [13] executive skills, trait and state of anxiety, fear of falls [12], fall risk [17, 18] and recurrent falls [19]. Two studies conducted an in-depth exploration of the TUG test and compared it to gait speed alone [12, 20]. Viccaro et al. [20] found that the TUG test is as good as gait speed alone for predicting future morbidity. However, its qualitative elements (sit-to-stand and turns) may predict mobility deterioration better than gait speed alone. Herman and colleagues [12] reported that the TUG is superior to other commonly used tests, such as the Berg Balance Scale and the Dynamic Gait Index, in its relationship to cognitive abilities and because it does not have a ceiling effect. Thus, while it is apparent that personality may be associated with mobility limitations, the association between personality and TUG single and dual-task is yet to be addressed. The objective of the current study was to explore the association between TUG and personality among community dwelling older adults.

## Methods

### Participants and procedure

Study procedures were approved by the University of Haifa Institutional Review Board. The participants were recruited by advertisements in community centres. Written informed consent for participation in the study was obtained from participants, after meeting the inclusion criteria. In this cross-sectional study, 85 community-dwelling older adults (mean age 74.39, SD-6.12) were recruited. Inclusion criteria were, 1) age 65 or older; 2) able to walk independently with or without assistive device; and 3) could speak, understand, and read Hebrew. Exclusion criteria were 1) a neurologic or musculoskeletal diagnosis, such as cerebral vascular accident, Parkinson’s disease, Alzheimer’s disease or multiple sclerosis; 2) severe orthopaedic restrictions such as acute back pain or a total hip or knee replacement; 3) significant hearing or vision loss.

### Measures

Each study visit was 1.5 h and included collection of (1) demographic characteristics as reported by the respondents, including age, gender, body mass index (BMI – in kg/m2), educational level (years of study), chronic disease (being diagnosed with cancer, hypertension, diabetes, or cardiovascular disease: yes/no), falls history (at least one) in the last year, based on self-report and physical activity (reflected by the number of weekly hours customarily engaged in physical activities). (2) Cognitive ability was assessed with the Montreal Cognitive Assessment (MoCA) [21], which covers 10 cognitive domains using rapid, sensitive, and easy-to-administer cognitive tasks. Using a cut-off score of 26, its sensitivity to detect minimal cognitive impairment (MCI) is 100 % and specificity is 87 % [21]. (3) Personality was assessed with the NEO-FFM, a short version of the original FFM [22]. It includes 60 statements on five domains of personality (twelve for each factor): Neuroticism, Extraversion, Openness, Agreeableness, and Conscientiousness- rated on a five-item Likert scale. The NEO-FFM subscales were totalled by the mean of each of twelve statements [22]. Internal consistency ranges from *α* = .60 to *α* = .86 and construct validity correlations with the original FFM range from *r* = .76 to *r* = .91 [22]. Several studies have published normative data for the FFM in older populations [9–11, 23]. (4) In the Timed-Up and Go test (TUGST) [13] subjects were asked to stand up and walk a distance of 3 meters at a comfortable pace, turn, walk back and sit down. The time for test completion was measured with a stop-watch. The participants used their customary walking aids and were not permitted to use their hands while rising from the chair. (5) Timed-Up and Go with cognitive dual-task (TUGDT) [15]- this test has high inter-rater reliability (ICC = 0.98) and good test-retest reliability ranging between 0.56 and 0.98 in different studies [24, 25]. The original study that described the TUG psychometric properties provides 87 % sensitivity and sensitivity to correctly identify fallers and non-fallers [15]. However, more recently a meta-analysis revealed lower sensitivity of between 21 % and 44 %, and specificity between 64 % and 71 % [26]. The participants performed the TUG test with an added cognitive task of subtraction by three, starting at a random number from 100 to 250. The total time to complete the test and the number of correct answers were recorded.

### Statistical analysis

SPSS version 15.0 software was used to calculate ordinary least squares (OLS) regressions, to examine the associations between personality factors and single and dual task TUG.

Using G power software [27], the sample size (*n* = 85) restricted the number of independent factors to nine (five traits of the FFM and four covariates) [27]. Thus, in the first step the main effect variables of the FFM traits were entered. These five factors were entered simultaneously to isolate the unique effect of each domain. In the second step, the four covariates (age, MoCA, BMI, and falls history) were entered to the regression model. We based our choice of which of the potential covariants to be entered on their significant correlations with the criteria [28]. Additionally, following previous work on falls history as a strong covariant [26], we added this variable to the regression model.

## Results

### Subject characteristics

The inter-correlations, means and their standard deviations appear in Table 1. A total of 85 participants completed the study, 33 males and 52 females. Mean age was 74.39 (SD 6.12.) The mean BMI was 25.63 (SD 3.52) and the mean Montreal Cognitive Assessment (MoCA) score was 23.28 (SD 2.98). Of the 85 participants, 14 (16 %) reported at least one fall in the previous year. The TUGST and TUGDT correlation was found to be low (*r* = .09, ns). Of the FFM traits, only Extraversion was significantly associated with TUGST (*r* = -.32, *p* < .01). The associations of TUGDT with the FFM traits were insignificant. Of the control variables, TUGST was negatively associated with MoCA and education (*r* = -.31, *p* < .01; *r* = -.22, *p* < .05, respectively) and positively associated with BMI and age (*r* = .28, *p* < .05; *r* = .36, *p* < .01, respectively). TUGDT was significantly associated only with MoCA (*r* = .27, *p* < .05).

**Table 1:** Intercorrelations, Means and Standard Deviations (SD) of the Study Variables

Measure	1	2	3	4	5	6	7	8	9	10	11	12	13	14	15
1. TUGST	-	-	-	-	-	-	-	-	-	-	-	-	-	-	-
2. TUGDT	.09	-	-	-	-	-	-	-	-	-	-	-	-	-	-
3. Neuroticism	.19	.06	-	-	-	-	-	-	-	-	-	-	-	-	-
4. Extraversion	-.32**	.09	-.28**	-	-	-	-	-	-	-	-	-	-	-	-
5. Conscientiousness	-.18	-.19	-.26*	.34**	-	-	-	-	-	-	-	-	-	-	-
6. Openness	-.01	-.07	.06	.12	.06	-	-	-	-	-	-	-	-	-	-
7. Agreeableness	.143	.081	-.085	-.014	.006	.208*	-	-	-	-	-	-	-	-	-
8. MoCA	-.31**	.27*	-.13	.05	.11	.23*	.18		-	-	-	-	-	-	-
9. BMI	.28*	-.10	.17	.05	.10	-.10	-.03	-.28**							
10. Gender a	.03	.13	.12	.07	-.17	.071	.38**	.19	-.03	-	-	-	-	-	-
11. Physical activity b	-.03	.10	-.05	-.08	-.03	-.01	-.05	-.06	-.05	-.14	-	-	-	-	-
12. Age	.36**	.02	-.05	-.07	-.10	-.12	-.14	-.42**	.01	-.07	-.03	-	-	-	-
13. Education (in years)	-.22*	.11	-.26**	.05	.092	.30**	.20*	.51**	-.18	.10	-.11	-.31**	-	-	-
14. Falls history c	-.08	-.02	.04	-.01	-.18	.03	-.11	-.01	-.20	-.11	-.13	.14	-.03	-	-
15. Chronic disease d	.13	.03	.08	-.24*	.12	-.23*	-.04	-.14	.13*	-.35*	-.07	.24*	-.21*	.18	-
M	9.95	4.85	2.61	3.21	3.89	3.39	4.01	23.28	25.63	2.62	3.60	74.39	13.98	.14	.26
SD	2.90	2.06	.62	.57	.53	.47	.50	2.98	3.52	.49	2.90	6.12	2.66	.35	.44

Notes: n=85; a gender coded 1 = woman, 0 = man; b Falls history coded: 1= yes 0=no; Chronic disease coded 1=yes 0=no; MoCA- Montreal Cognitive Assessment; BMI- Body Mass Index; *p < .05; **p< .01

### The Association between TUG and Personality

Results of the OLS regression analyses testing the associations between the FFM and TUG are reported in Table 2. Extraversion was inversely associated with time to complete the TUGST (β = -.26, *p* < .05). Conscientiousness was negatively associated with TUGDT (β = -.24, *p* < .05). The other FFM traits, Neuroticism, Agreeableness and Openness, were not found to be associated with TUG in single nor dual task.

**Table 2:** Results of the OLS regression analyses testing the association between the Five Factor Model and the Timed Up & Go (TUG) single task (ST) and dual task (DT)

	TUGST (n=85)			TUGDT (n=85)		
	B	SEB	β	B	SEB	β
Neuroticism	.55	.51	.12	.19	.39	.05
Extraversion	-1.30**	.56	-.26	.62	.42	.17
Conscientiousness	-.50	.58	-.09	-.91*	.44	-.24
Openness	-.35	.65	-.06	-.44	.49	-.10
Agreeableness	.90	.61	.16	.58	.46	.14
Covariates						
MoCA	-.13	.10	-.14	.27**	.08	.39
BMI	.22**	.09	.27	-.03	.07	-.04
Age	.15**	.05	.32	.07	.04	.19
Falls history	-.44	.78	-.05	-.44	.63	-.07

Notes: B and β represent the non standardized and standardized partial regression coefficients, respectively; SEB stands for the standard error of the former; MoCA- Montreal Cognitive Assessment; BMI- Body Mass Index; *p < .05; **p< .01.

## Discussion

The current study was the first to explore the association between all five personality traits that comprise the FFM and the most frequently used test for mobility evaluation, the TUG. We observed that persons with high Extraversion completed the TUGST test in less time than those who did not measure as high on this trait. Similarly, people with high Conscientiousness completed the TUGDT more quickly. These findings persisted even after controlling for several potential confounders, such as cognitive status, age, BMI, and falls history. Other traits of the FFM did not show an association with TUG in single- or dual-task in the current study. Time to complete the TUG was associated with an increased number of falls among elderly individuals [15]. Our findings agree with those of previous studies that examined an association between Conscientiousness and gait speed among the elderly [9], and an association between Extraversion and reduced risk of disability in older age [6]. However, the current study is the first to test the association with the full range of FFM dimensions and an objective measure of mobility among the elderly.

Extraversion encompasses the tendency toward positive mood, sociability, and activity [29]. The tendency to be friendly toward others and to usually have positive emotions and attitudes may indicate a predisposition for engaging in a broad range of social behaviours that include an active, busy, or engaged lifestyle, which promotes better physical health [30], apparently including decreased risk of falls. In the current study, high extroversion was associated with TUG as a single task, but not as a dual task. These findings contradict a new study that found that high Extroversion and low Neuroticism are associated with better ability to divide attention while walking [23]. This discrepancy may be associated with the fact that LeMonda and colleagues [23] used in their study only two of the five factor of the FFM. In our study, the five factors were used simultaneously to isolate the unique effect of each domain. Additionally, while we used the valid dimensional classification (i.e., using the five factors as continuous), LeMonda and colleagues [23] used a categorical classification which may not be adequate. Another explanation may be associated with the different tasks that were explored: we used a short mobility test that incorporates other mobility aspects, such as sit to stand and turns. Future study should further explore the effect of Extroversion on mobility.

Conscientiousness is considered one of the most significant determinates of positive health outcomes [31]. Older adults with high Conscientiousness reported fewer functional limitations, higher functioning in instrumental daily living activities and decreased risk of developing disability [9]. The findings from the current study add to this information by supporting the protective effect of Conscientiousness against mobility deterioration among the elderly, as expressed by the dual-task TUG test. The TUG test was found to be a significant predictor of ADL disability at 6 [32] and 12 months after a fall [20], as well as of future falls [17, 18]. An earlier study demonstrated positive association between gait speed and Conscientiousness [9]. Our findings further support these reports by demonstrating an association with the TUG that includes other aspects of mobility, such as turns and sit-to-stand.

The relationships between Conscientiousness and mobility are complex and several mechanisms are likely to be involved. In general, people with high Conscientiousness are likely to be engaged in regular physical activities [33]. However, in the current study we did not demonstrate a mediating role of physical activity, probably due to the high functioning status that characterized our study’s sample. Previous studies demonstrated that people who are characterized by high Conscientiousness are more engaged in physical activities [33], which positively affects mobility. People with high Conscientiousness may engage in more social support behaviours [34], which contribute to cognitive reserve [35] and are expressed as better performance in dual-task TUG. Conscientiousness has been frequently described as associated with better physiological characteristics such as lower body mass index, lower inflammation biomarkers and higher longevity (see review of Chapman et al., 2011) [5].

No relationships between Agreeableness, Neuroticism, and Openness to performance on TUG as single or as a dual-task were found. No association between Openness and Agreeableness to mobility was previously demonstrated in other studies [9, 11], probably due to the fact that these traits are less relevant for physical activity and mobility as evaluated in this context. In addition, in a recent study the combination of high Neuroticism and low Extroversion was associated with lower ability to conduct walking with DT [23]. In the current study high no association to Neuroticism was found and extroversion was only associated with better performance on TUG as a single task, but not as a DT. The discrepancy between these findings may stem from different methodologies for evaluating mobility or for quantifying personality traits from the FFM.

The findings reported here should be interpreted with caution, due to several limitations of the study. The sample was small and included relatively high- functioning, older adults. However, it is possible that these relationships would be manifested more strongly among frail, elderly individuals. The cross-sectional design limited the ability to infer causality. In addition, this study relied on a performance based test for mobility (TUG) and not on mobility in a daily life. However, similar design was implemented by others [23, 36]. The ability of the TUG test to predict falls for a single task was recently challenged [26], however, in this study DT was added, and this addition may improve the predictive value of the TUG [26] test. Future studies should confirm these findings in real life situations.

## Conclusions

Findings from this study highlight the relationship between personality and the TUG test. Specifically, older adults with high Extraversion completed the TUGST test more quickly than those who had lower measures of this trait and, people with high Conscientiousness completed the TUGDT tests more quickly than those who had lower measures of this trait. These findings may contribute to early identification of older adults at higher risk from mobility limitations and falls, and to developing personality-tailored interventions for fall prevention. The findings have clinical implications in that interventions aimed at detecting and improving mobility deterioration among the elderly could be more effective if designed to consider personality traits. Addressing risk factors for falls is the main strategy for fall prevention [14]. As such, identification of personality traits that are associated with a greater risk of developing mobility limitations may help to initiate prevention programmes to reduce mobility limitations. For example, people who score lower in Conscientiousness and Extroversion may benefit from early detection and intervention and may gain from closer personal support than those who are characterized by high scores on these traits. Future research should investigate possible longitudinal associations between personality and mobility and address frail populations, as well.

### Availability of supporting data

The data are available upon request.

## Abbreviations

TUG: timed-up and go
TUGST: timed-up and go as single task
TUGDT: timed-up and go with dual-task
